# Bilateral maxillary brown tumors in a patient with primary hyperparathyroidism: Report of a rare entity and review of literature

**DOI:** 10.4103/0973-029X.80027

**Published:** 2011

**Authors:** N Soundarya, P Sharada, Nilima Prakash, GL Pradeep

**Affiliations:** *Department of Oral and Maxillofacial Pathology, M. R. Ambedkar Dental College, Cline road, Cooke town, Bangalore, India*; 1 *AECS Maaruti College of Dental Sciences and Research centre, Bangalore, India*; 2 *MGV’s KBH Dental College and Hospital, Nashik, India*

**Keywords:** Brown tumor, hyperparathyroidism, maxilla

## Abstract

Brown tumors are erosive bony lesions caused by rapid osteoclastic activity and peritrabecular fibrosis due to hyperparathyroidism, resulting in a local destructive phenomenon. The classical “brown tumor” is commonly seen in ends of long bones, the pelvis and ribs. Facial involvement is rare and, when present, usually involves the mandible. We report a case of 60-year-old male with a rare initial presentation of primary hyperparathyroidism with bilateral maxillary brown tumors. The present case represents the third report of the bilateral maxillary brown tumors in a patient with primary hyperparathyroidism. Differential diagnosis is important for the right treatment choice. It should exclude other giant cell lesions that affect the maxillae.

## INTRODUCTION

Brown tumor is an osseous lesion that develops in bones affected by primary or secondary hyperparathyroidism, as a component of a metabolic bone disease known as osteitis fibrosa, cystica generalisata or Recklinghausen’s disease of bone.[1] The name “tumor” is a misnomer because the lesion, although invasive in some instances, does not have a neoplastic potential and should be differentiated from true giant cell tumors of bone.[2] The common sites of brown tumors are the long bones, pelvic girdle, clavicle, ribs and the mandible. Tumors involving the maxillae are rare.[3]

We report the case of a 60-year-old male who presented with brown tumors of both maxillae. The present case represents the third report of bilateral maxillary brown tumors in a patient with primary hyperparathyroidism.[3] The report highlights the need to consider primary hyperparathyroidism in the differential diagnosis of tumorous lesions of the maxillae.

## CASE REPORT

**Figure 1:**
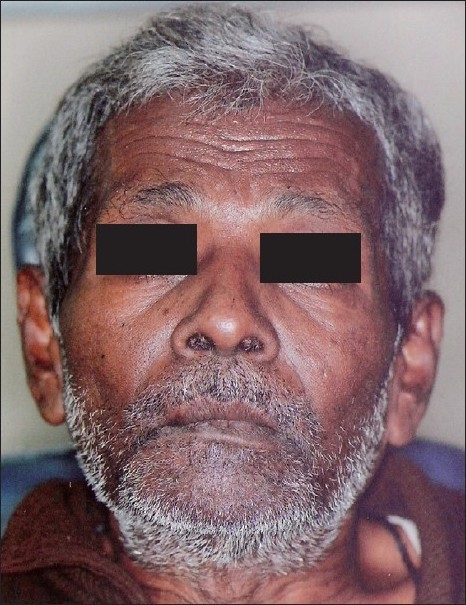
A diffuse swelling in the left infra-orbital and zygomatic region

**Figure 2:**
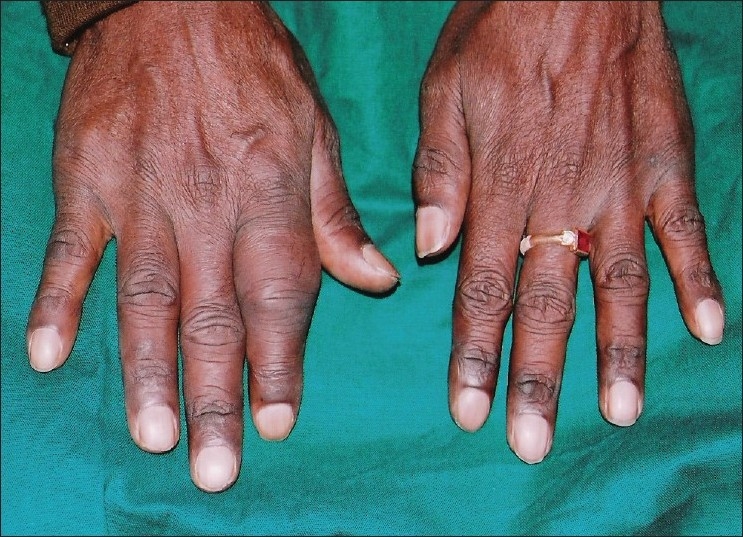
Swelling on the right index finger

**Figure 3:**
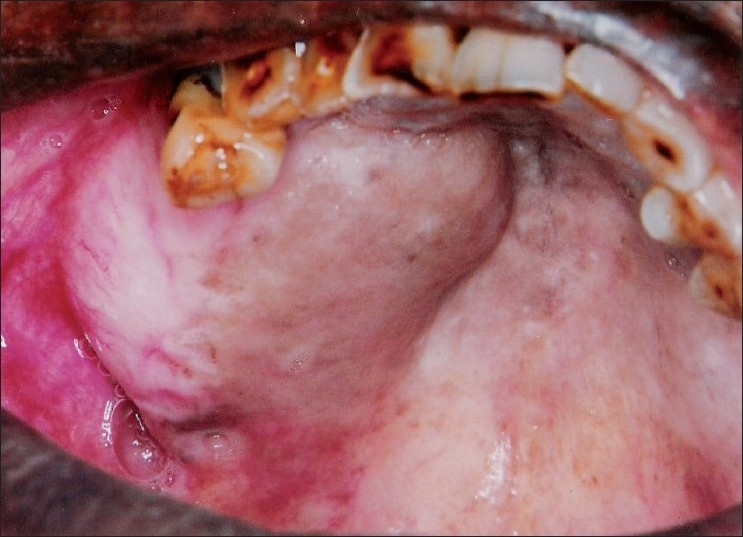
Swelling of the alveolar ridge on the right maxilla extending up to the midline of the palate

**Figure 4:**
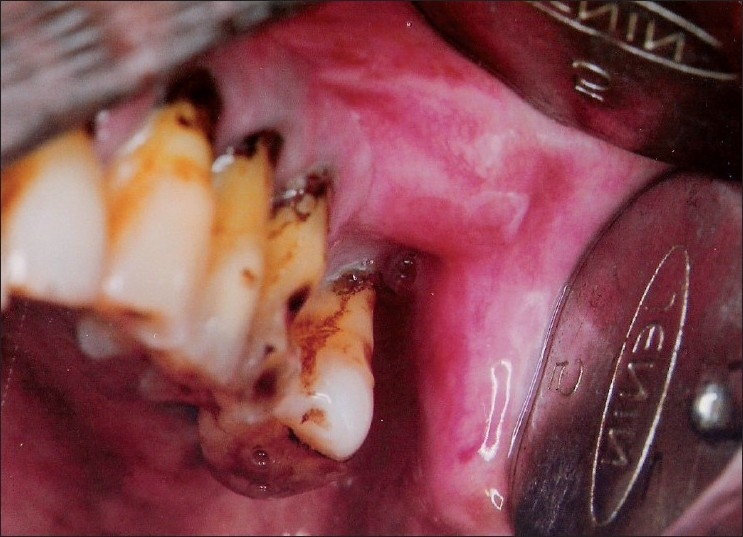
Swelling was present on the left maxillary sulcus in relation to 24, 25, 26

**Figure 5:**
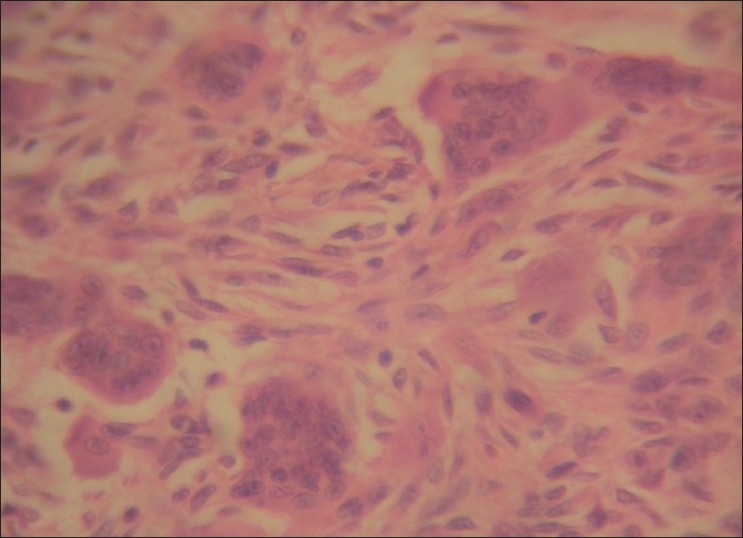
Multinucleated giant cells scattered in cellular fibroblastic tissue background (H and E, 40×)

A 60-year-old male reported to our hospital with progressive swelling in the upper left and right jaw since 6 months. The patient noticed a swelling on the right index finger 7 months back. The patient complained of pain in the jaw swellings since 1 week. The past medical history and family history of the patient was noncontributory. On extraoral examination, a diffuse swelling in the left infraorbital and zygomatic region was observed [Figure 1]. A swelling was noted on the right index finger [Figure 2]. Intraorally, a well-defined swelling was seen in relation to the alveolar ridge, extending from buccal sulcus to the midline of the palate on the right side and anteriorly from premolar region to the tuberosity region [Figure 3]. The lesion was soft in consistency and tender on palpation. Another swelling was present on the left side of the maxilla, in the sulcus region in relation to 24, 25, 26 [Figure 4]. Grade II mobility of 25, 26 were noted. Severe cervical abrasion of 12, 13, 25, and generalized gingival recession were observed. On radiographic examination, a well-defined lytic lesion causing expansion of the affected maxilla was noted. X-rays of the hands showed subperiosteal resorption, particularly of the phalanges of the right index finger. Blood chemistry findings included the following: calcium level 7.7 mg/dL, phosphorus 2.9 mg/dL, alkaline phosphatase activity 747 U/L (normal range 66-220 U/L) and parathyroid hormone (PTH) 121 pg/mL (normal range 15-50 pg/mL). Serum urea (45 mg/dL) and creatinine (1.1 mg/dL) levels were normal. The patient was not willing to undergo investigations to determine the cause of primary hyperparathyroidism. An incisional biopsy of the right-sided lesion was performed. Microscopic examination revealed multinucleated giant cells scattered in a cellular fibroblastic tissue background. Areas of hemorrhage and spicules of woven bone were seen [Figure 5]. These findings were consistent with a giant cell lesion. Complete resection of the tumor was performed. Grossly, the mass was dark reddish brown in color. The microscopic findings were similar to the incisional biopsy specimen. Brown tumor and giant cell tumor were suggested as possible diagnosis. The final diagnosis of brown tumor was confirmed based on the above laboratory data and characteristic histopathologic findings.

## DISCUSSION

Sylvanus (1743) was the first to diagnose hyperparathyroidism. Recklinghausen (1891) is credited with the first description of the associated bone changes known as osteitis fibrosa cystica.[4]

Primary hyperparathyroidism is the uncontrolled production of PTH, usually as a result of a parathyroid adenoma or hyperplasia of glands. Infrequently, a parathyroid carcinoma may be the cause.[5] The bone changes result from the direct effect of PTH on bone, causing the conversion of potentially osteogenic cells from osteoblasts to osteoclasts, with bone resorption exceeding the formation of new osseous tissue.[4]

The reported prevalence of brown tumors is 0.1%.[6] Brown tumors arise secondary to both primary and secondary hyperparathyroidism, and have been reported to occur in 4.5% of patients with primary hyperparathyroidism and in 1.5–1.7% of those with secondary disease.[7] The disease can manifest at any age, but it is more common among persons older than 50 years, and is three times more common in women than in men.[8] Brown tumors can occur in monoostotic and polyostotic forms. Brown tumors commonly affect the mandible, clavicle, ribs, pelvis, and femur.[9] In the craniofacial bone, the involvement of maxilla, palate, temporal bone, nasal cavity, orbital bone, and paranasal sinuses has also been reported.[10]

Dental changes reported in association with hyperparathyroidism include abnormally large pulp chambers in developing teeth. In erupted teeth, the pulp chambers may appear abnormally large in young patients; however, in adults, it may be narrower than expected in correlation with the patient’s age. The pulp space narrowing was evident in patients with end-stage renal disease.[11] Amelogenesis imperfecta has been reported in patients with secondary hyperparathyroidism.[12]

Bilateral maxillary lesions have been reported earlier by Khochtali, Ach, Jlidi, Bouhaouala *et al*. (1991) and Felix Jebasinghin (2008). Most patients with hyperparathyroidism are asymptomatic. Hypercalcemia is often discovered incidentally during routine laboratory testing; hypophosphatemia and increased alkaline phosphatase levels in blood may also be seen.[8]

Radiographically, brown tumors appear as well-defined marginated expansile lytic lesions and may cause cortical expansion. Concurrent bone changes associated with hyperparathyroidism, such as generalized demineralization of the medullary bones of the jaw and loss of lamina dura around the roots of teeth, can help differentiate brown tumors from other processes.[9,10]

Grossly, a brown tumor appears as a mass with partly cystic and partly solid areas. Microscopically, brown tumors are characterized by intensely vascular fibroblastic stroma serving as a background for numerous osteoclast-like multinucleated giant cells.[1] Cysts develop as a result of intraosseous bleeding and tissue degeneration. The cystic spaces are filled up by clusters of giant cells, hemosiderin-laden macrophages and plump fibroblasts. The presence of hemorrhage, hemosiderin and hypervascularity leads to the brownish color, and thus the name.[3] The presence of the giant osteoclasts in the lesions along with plump fibroblasts leads to confusion of the tumor with other jaw lesions that contain giant cells.[13] Histological features alone cannot establish a certain diagnosis of a brown tumor. However, a clinical history of more widespread skeletal involvement, pathological fractures and renal stones may suggest the presence of primary hyperparathyroidism. The diagnosis is readily confirmed by establishing elevated serum calcium and PTH levels. There is a familial form of hyperparathyroidism associated with jaw tumors in which the histology of the jaw tumor shows an ossifying fibroma. This can be readily distinguished from brown tumors on histological grounds.[14]

Treatment of hyperparathyroidism is the first step in the management of brown tumor. After appropriate medical or surgical treatment of the underlying endocrine abnormality, brown tumor may regress, and almost all radiographic changes tend to return to normal.[9] An occasional large brown tumor or persistent deformity may require operative intervention.[3]

## CONCLUSION

Brown tumors of the jaw may commonly involve the mandible and rarely involve the maxilla in association with primary hyperparathyroidism. The presence of underlying primary hyperparathyroidism should be sought in all unexplained mandibular and maxillary lesions.
